# Ciprofloxacin-Resistant *Campylobacter* spp. in Retail Chicken, Western Canada

**DOI:** 10.3201/eid1907.111417

**Published:** 2013-07

**Authors:** Agnes Agunos, David Léger, Brent P. Avery, E. Jane Parmley, Anne Deckert, Carolee A. Carson, Lucie Dutil

**Affiliations:** Public Health Agency of Canada, Guelph, Ontario, Canada (A. Agunos, D. Léger, B.P. Avery, E.J. Parmley, A. Deckert, C.A. Carson);; Public Health Agency of Canada, Saint-Hyacinthe, Québec, Canada (L. Dutil)

**Keywords:** *Campylobacter*, antimicrobial resistance, fluoroquinolone, surveillance, retail chicken, bacteria, Canada

## Abstract

During 2005–2010, the Canadian Integrated Program for Antimicrobial Resistance Surveillance identified increased prevalence of ciprofloxacin (a fluororquinolone) resistance among *Campylobacter* isolates from retail chicken in British Columbia (4%–17%) and Saskatchewan (6%–11%), Canada. Fluoroquinolones are critically important to human medicine and are not labeled for use in poultry in Canada.

Human campylobacteriosis, a notifiable disease in Canada, is the most common cause of bacterial enteric infections among persons in Canada; in 2005, the incidence rate of campylobacteriosis was 30.9 cases per 100,000 population (*1*). In chickens, *Campylobacter* spp. are not clinically relevant; however, the presence of these bacteria in poultry represents a potential threat to public health (*2*).

Ciprofloxacin, a fluoroquinolone antimicrobial drug, is indicated for the treatment of respiratory, urinary, skin, and bone/joint infections and gastroenteritis in adults (*3*). In 2008 in Canada, fluoroquinolones were the fourth most frequently dispensed class of antimicrobial drug (dispensed for oral use by retail pharmacists; www.phac-aspc.gc.ca/cipars-picra/2008/4-eng.php#Hum0). A study investigating antimicrobial drug use and resistance in 2 health units in Ontario found that ciprofloxacin was the antimicrobial drug most frequently used to treat human campylobacteriosis (*4*). Fluoroquinolones are considered “critically” or “very” important to human medicine by the World Health Organization (*5*) and the Veterinary Drugs Directorate (VDD), Health Canada (*6*). The veterinary fluoroquinolones enrofloxacin and danofloxacin are VDD Category I antimicrobial drugs labeled for use in companion animals and beef cattle, but they are not labeled for use in poultry. The VDD has established a policy recommending against the extra-label use of Category I antimicrobial drugs in food-producing animals (*7*); however, Canada does not have legislation restricting this extra-label use.

## The Study

The Canadian Integrated Program for Antimicrobial Resistance Surveillance (CIPARS), Public Health Agency of Canada, collects samples of fresh chicken, beef, and pork from retail outlets in British Columbia, Saskatchewan, Ontario, Québec, and Maritimes (New Brunswick, Nova Scotia, and Prince Edward Island) on a routine basis and cultures them for select bacteria. Retail food samples best reflect the level of consumer exposure to drug-resistant foodborne bacteria. Methods used for sample collection, culture, and antimicrobial drug-susceptibility testing are described in the CIPARS annual reports (www.phac-aspc.gc.ca/cipars-picra/2008/6-eng.php#Ant).

Up to and including 2010, *Campylobacter* spp. were isolated from retail chicken meat samples in the provinces of British Columbia (since 2007, 225 isolates/536 samples [42%]), Saskatchewan (since 2005, 276/884 [31%]), Ontario (since 2003, 845/2288 [37%]), Québec (since 2003, 683/2215 [31%]), and the Maritimes (since 2008, 117/444 [26%]) (Table). Temporal trends in ciprofloxacin resistance among *Campylobacter* spp. isolates are shown in the Figure. Since initiation of surveillance, prevalence of ciprofloxacin resistance has significantly increased in British Columbia and Saskatchewan. The highest recovery of ciprofloxacin-resistant *Campylobacter* spp. was in British Columbia in 2009 (28.6%, 22/77), when recovery of all *Campylobacter* spp. (52.7%, 77/146) was also highest. 

**Table:** *Campylobacter *spp. isolated from retail chicken in Canada, by province, 2003–2010*

Province	2003	2004	2005	2006	2007	2008	2009	2010
British Columbia								
Samples collected, no.					80	145	146	165
*Campylobacter* spp. isolates, no. (%)					28 (35.0)	50 (34.5)	77 (52.7)	70 (42.4)
Ciprofloxacin-resistant isolates, no. (%)					1 (3.6)	4 (8.0)	22 (28.6)	12 (17.1)
Saskatchewan								
Samples collected, no.			145	155	141	161	150	132
*Campylobacter* spp. isolates, no. (%)			52 (35.9)	51 (32.9)	49 (34.8)	40 (24.8)	48 (32.0)	36 (27.3)
Ciprofloxacin-resistant isolates, no. (%)			3 (5.8)	1 (2.0)	3 (6.1)	4 (10)	7 (14.6)	4 (11.1)
Ontario								
Samples collected, no.	166	316	303	312	320	311	328	232
*Campylobacter* spp. isolates, no. (%)	78 (47.0)	140 (44.3)	120 (39.6)	105 (33.7)	117 (36.6)	120 (38.6)	101 (30.8)	64 (27.6)
Ciprofloxacin-resistant isolates, no. (%)	3 (3.8)	3 (2.1)	3 (2.5)	3 (2.9)	1 (0.9)	5 (4.2)	1 (1.0)	3 (4.7)
Québec								
Samples collected, no.	170	322	299	288	287	287	266	296
*Campylobacter* spp. isolates, no. (%)	94 (55.3)	158 (49.1)	103 (34.4)	100 (34.7)	59 (20.6)	54 (18.8)	52 (19.5)	63 (21.3)
Ciprofloxacin-resistant isolates, no. (%)	3 (3.2)	4 (2.5)	2 (1.9)	2 (2.0)	8 (13.6)	0	0	1 (1.6)
Maritimes								
Samples collected, no.						55	199	190
*Campylobacter* spp. isolates, no. (%)						2 (3.6)	47 (23.6)	68 (35.8)
Ciprofloxacin-resistant isolates, no. (%)						0	2 (4.3)	3 (4.4)

*Blank cells indicate not applicable (no samples collected or tested).

**Figure:**
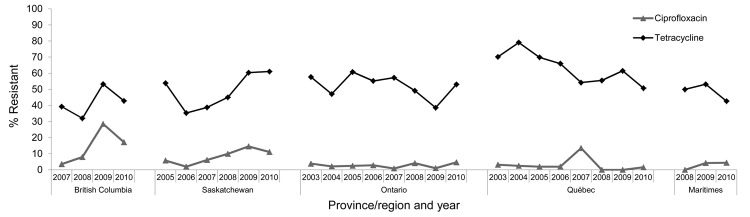
Temporal trends in ciprofloxacin and tetracycline resistance among *Campylobacter* isolates from chicken, Canada, 2003–2010. Data for 2003 to 2009 were published in the CIPARS 2009 Preliminary Report (www.phac-aspc.gc.ca/cipars-picra/2009/1-eng.php#fig_21). Data for 2010 were published in the CIPARS 2010 Short Report (can be requested at www.phac-aspc.gc.ca/cipars-picra/pubs-eng.php). CIPARS, Canadian Integrated Program for Antimicrobial Resistance Surveillance, Public Health Agency of Canada.

Significant differences in prevalence between 2 periods were assessed by using χ^2^ tests. The increased trend in British Columbia seems more abrupt, especially from 2008 to 2009, relative to Saskatchewan, where the increase is relatively more gradual. Combined data from 2009–2010 showed that 23% (34/147) of *Campylobacter* spp. isolates from British Columbia and 13% (11/84) of isolates from Saskatchewan were resistant to ciprofloxacin, compared with 6% (5/78, p = 0.002) and 6% (11/192, p = 0.04) of isolates resistant to ciprofloxacin in combined data before 2009 in British Columbia (2007–2008) and Saskatchewan (2005–2008), respectively. By comparison, in 2009–2010, the overall prevalence of ciprofloxacin-resistant *Campylobacter* spp. from retail chicken collected in Ontario, Québec, and the Maritimes has remained <3% (10/395), significantly lower (p<0.001) than the overall prevalence in British Columbia and Saskatchewan (19%, 45/231). The current prevalence of ciprofloxacin resistance in British Columbia and Saskatchewan is also higher than that reported by studies of antimicrobial drug resistance in *Campylobacter* spp. in Ontario (*8*) and Québec (*9*).

Several factors contributing to the emergence of ciprofloxacin resistance have been hypothesized and include antimicrobial drug use in broiler breeder and broiler chickens and importation of poultry products. Data for antimicrobial drug use in the poultry industry are not currently available. Fluoroquinolone use can select for ciprofloxacin resistance and result in the emergence and persistence of resistant *Campylobacter* spp. (*10*)*.* Evidence indicates vertical transmission of fluoroquinolone-resistant *Campylobacter *spp. (*11*). Surveillance data from the United States showed the persistence of ciprofloxacin resistance, despite a 2005 ban on fluoroquinolone use in chickens (*12*). Ciprofloxacin resistance also persisted in broilers raised in Denmark after fluoroquinolone use decreased in 2006 (*13*). Current use of ciprofloxacin in US poultry is unknown. However, in Denmark, use in few broiler breeders has been reported (*13*)*.* It is plausible that fluoroquinolone use in breeder and broiler chickens could explain the generation and maintenance of ciprofloxacin resistance.

Another antimicrobial drug–use practice might play a role in ciprofloxacin resistance. Tetracyclines are labeled for use in broiler chickens. Across the regions sampled by CIPARS, a high proportion (64%) of ciprofloxacin-resistant *Campylobacter* isolates were also resistant to tetracycline. Although the prevalence rates for ciprofloxacin and tetracycline resistance were quite different, the resistance trends in British Columbia and Saskatchewan were strikingly similar in shape. Tetracycline resistance was significantly associated with ciprofloxacin resistance in isolates from British Columbia and Saskatchewan (odds ratio 3.04; 95% CI 1.70–5.48; p<0.001) but not in those from the eastern part of the country (odds ratio 0.99; 95% CI 0.54–1.38; p = 0.951). A similar phenomenon has recently been noted among *Campylobacter* spp. recovered from domestically raised and imported broiler chickens in Denmark (*13*). We currently have no explanation for this observation; *gyrA* mutation and overexpression of the multidrug-resistant efflux pump *CmeABC* (*14*), as a result of exposure to ciprofloxacin and ciprofloxacin/tetracycline, respectively, might have played a role. Molecular investigations of organisms isolated from retail chicken by CIPARS have yet to be conducted.

Another factor to consider is the importation of poultry products. CIPARS collects raw, fresh (never frozen) chicken primarily produced and distributed in Canada. It is possible that a small proportion of the fresh retail meat sampled originated in other countries, but the volume of imported meat (≈75 tonnes [167 million pounds] or 7.5% of the previous year’s production; [*15*]) is unlikely to explain the regional increase in *Campylobacter* spp. resistance. Furthermore, importation of meat is not restricted to only British Columbia and Saskatchewan (www.international.gc.ca/controls-controles/prod/agri/chicken-poulet/index.aspx?menu_id = 26&view = d). According to trade agreements, ≈141 million chicks/eggs (≈21.1% of the previous year’s grown broiler production) are imported annually from the United States. These imported broiler chicks/eggs might be an additional source of drug-resistant subtype introduction into the Canadian poultry production system. To evaluate the prevalence of resistance in domestically raised flocks, the CIPARS abattoir component added *Campylobacter* spp. surveillance in chickens in 2010. From this surveillance, 4/111(4%) isolates from domestically raised chicken ceca were resistant to ciprofloxacin, 3 from British Columbia, and 1 from the Maritimes, confirming the potential domestic origin of these strains in retail chicken.

## Conclusion

The Public Health Agency of Canada is concerned by the emergence of resistance to ciprofloxacin, which is critically important for treatment of *Campylobacter *spp. infection in humans. Extra-label use of fluoroquinolones in the broiler breeder or broiler chicken sectors might have contributed to the emergence of this resistance. The role of importation of poultry products as a potential source of resistant strains requires further investigation. The broiler industry is collaborating with CIPARS to create a farm surveillance program, which would capture data on antimicrobial drug use and resistance.

Veterinary fluoroquinolones are not labeled for use in poultry in Canada. The VDD policy recommends against the extra-label use of Category I antimicrobial drugs in food-producing animals. More research is required to assess mechanisms responsible for the trends observed for tetracycline and ciprofloxacin resistance. Furthermore, genotyping of isolates from humans and chickens (retail and/or abattoir) from British Columbia and Saskatchewan is required to determine if strains are epidemiologically related.
